# The effect of thoracolumbosacral orthosis on scoliosis progression and chest deformity in children with type 1 spinal muscular atrophy: A randomized controlled trial

**DOI:** 10.1371/journal.pone.0323341

**Published:** 2025-09-15

**Authors:** Emre Dansuk, Ayşe Nur Tunalı Van Den Berg, Görkem Ata, Seval Kutluturk Yıkılmaz, Sedat Oktem

**Affiliations:** 1 Department of Physiotherapy and Rehabilitation, Faculty of Health Sciences, Istanbul Medipol University, Istanbul, Turkey; 2 Department of Physiotherapy and Rehabilitation, Faculty of Hamidiye Health Sciences, University of Health Sciences, Istanbul, Turkey; 3 Department of Pediatric Pulmonary Diseases, Faculty of Medicine, Istanbul Medipol University, Istanbul, Turkey; Iran University of Medical Sciences, IRAN, ISLAMIC REPUBLIC OF

## Abstract

**Background:**

Type 1 spinal muscular atrophy (SMA) is an autosomal recessive neuromuscular disease characterized by severe muscle weakness, which results in progressive spinal and chest deformities. This study aims to evaluate the effect of thoracolumbosacral orthosis (TLSO) use along with pulmonary care (PC), individualized pulmonary rehabilitation (IPR), and individualized trunk exercises (ITE) in children with Type 1 SMA.

**Methods:**

The study enrolled 24 children with Type 1 SMA aged 2–6 years, with a scoliosis angle of 20°–40°. Participants were randomly assigned into two groups using a stratified randomization method: Group 1 (PC, IPR, ITE) and Group 2 (PC, IPR, ITE & TLSO). All participants underwent an 8-week treatment program. Pre- and post-treatment assessments included scoliosis progression measured by the Cobb angle, chest deformity evaluated through the basal upper-lower chest wall ratio and the Supine Angle of Trunk Rotation Test (SATR), and motor function levels assessed using the Children’s Hospital of Philadelphia Infant Test of Neuromuscular Disorders (CHOP INTEND).

**Results:**

Significant improvements were observed in Cobb angle, bell-shaped chest deformity, and motor function in both groups (p < 0.05). Group 2 demonstrated greater improvements in effect size (ES) across all evaluation parameters. Compared to Group 1, Group 2 showed superior improvement in Cobb angle (ES = 3.98), basal upper-lower chest wall ratio (ES = 5.00), SATRL (lower) (ES = 2.55), SATRU (upper) (ES = 1.64), and CHOP INTEND (ES = 1.23) (p < 0.05).

**Conclusions:**

This study is the first to demonstrate that the combination of PC, IPR, ITE, and TLSO yields superior clinical outcomes in children with Type 1 SMA. The findings support current recommendations for TLSO use in patients with a Cobb angle >20°, and emphasize the potential benefits of early, proactive orthotic intervention when integrated with mobilization, trunk, and pulmonary exercise programs in managing scoliosis in this population. However, limitations such as the small sample size and short follow-up period underscore the need for larger and longer-term studies to confirm these findings. **Trial Registiration:** NCT05878418

## Introduction

Spinal muscular atrophy (SMA) is an autosomal recessive disorder with an estimated incidence of 1 in 10,000 live births [1,2]. It is caused by a mutation in the *SMN1* gene, resulting in a deficiency of the survival motor neuron (SMN) protein, which leads to spinal motor neuron degeneration and progressive muscle weakness [3,4]. The severity of SMA is inversely correlated with the number of copies of the centromeric form of the *SMN2* gene [5]. Based on clinical features, age of onset, the highest achieved motor function, and expected lifespan, SMA is classified into five subtypes (SMA types 0–4) [6]. Type 1 SMA, the most severe form aside from the fatal neonatal Type 0, accounts for approximately 60% of all diagnosed SMA cases [2,7]. If left untreated, infants with Type 1 SMA do not attain independent sitting, and respiratory failure typically leads to death before the age of two [8]. In recent years, advances in pharmacological treatments have extended survival and improved symptoms in Type 1 SMA patients [9,10]. As these patients live longer, scoliosis and chest deformities have emerged as prevalent complications in the later stages of life [9].

Scoliosis, defined as a Cobb angle of >10° on spinal X-ray, varies in severity and is classified as mild (10–20°), moderate (20–40°), or severe (>40°) [10]. In SMA patients, scoliosis development is primarily attributed to axial muscle weakness and the inability to provide sufficient support for the growing spine [11]. Consequently, these patients often present with hypotonic spinal curvature, predisposing them to early-onset, progressive, and severe scoliosis [11,12]. Historically, scoliosis has been a major concern in patients with Type 2 SMA. However, in Type 1 SMA, spinal deformities were rarely observed due to poor prognosis and high early mortality rates [13,14]. With the advent of novel pharmacological and gene therapies that enhance motor and respiratory function while extending survival, the prevalence of scoliosis has increasingly shifted from Type 2 SMA to children with Type 1 SMA [15,16].

Spinal deformities are frequently associated with chest deformities, with a bell-shaped chest being a characteristic feature of Type 1 SMA. Weakness of the intercostal muscles restricts thoracic expansion and leads to the inward collapse of the ribs during inspiration [18,19]. This abnormal breathing pattern results in upper lung lobe atelectasis and impaired chest wall development, ultimately contributing to the formation of a bell-shaped chest deformity [9,20].

The increasing prevalence of scoliosis and scoliosis-related symptoms in patients with Type 1 SMA highlights the growing need for physiotherapy interventions [9,21]. A key area of research focuses on evaluating the effectiveness of pre-surgical spinal orthoses and their potential role in slowing disease progression [22]. In the literature, spinal orthoses are frequently recommended to support the hypotonic trunk and manage scoliosis exceeding 20°, particularly in children with SMA who exhibit significant growth potential [22–24]. However, despite these recommendations, there is no consensus on the optimal type of thoracolumbosacral orthosis (TLSO) or its application protocol [9]. Moreover, the natural progression of SMA is characterized not only by upper and lower extremity muscle weakness but also by severe trunk weakness, which predisposes patients to early-onset scoliosis [25]. Given the shared neuromuscular impairments, a study on children with Duchenne muscular dystrophy suggested that a trunk-focused exercise program could be beneficial in improving trunk control in pediatric patients [26].

To our knowledge, a limited number of studies have examined the use of TLSO in patients with SMA, and no study has specifically investigated the combined application of TLSO and trunk exercises in children with Type 1 SMA. Therefore, this study aims to assess the impact of TLSO and trunk exercises on Cobb angle, bell-shaped chest deformity, and motor function in children with Type 1 SMA. We hypothesize that this combined approach will enhance scoliosis progression, chest deformity, and motor function outcomes.

## Methods

### Study design and participants

This stratified, single-blind (evaluator), parallel-group randomized controlled trial (1:1 allocation ratio) was conducted at Istanbul Medipol University Hospital. Ethical approval was granted by the Non-Interventional Ethics Committee of Istanbul Medipol University (Approval Number: E-10840098-772.02-200). Both written and verbal informed consent were obtained from each participant’s family, ensuring adherence to the ethical principles outlined in the Declaration of Helsinki. The individual pictured in Fig 3 has provided written informed consent (as outlined in PLOS consent form) to publish their image alongside the manuscript. Additionally, the study was registered on ClinicalTrials.gov (NCT05878418), and the authors confirm that all active and related trials involving this intervention have been properly registered. The study commenced on October 11, 2023, and was completed on September 10, 2024.

**Fig 1:**
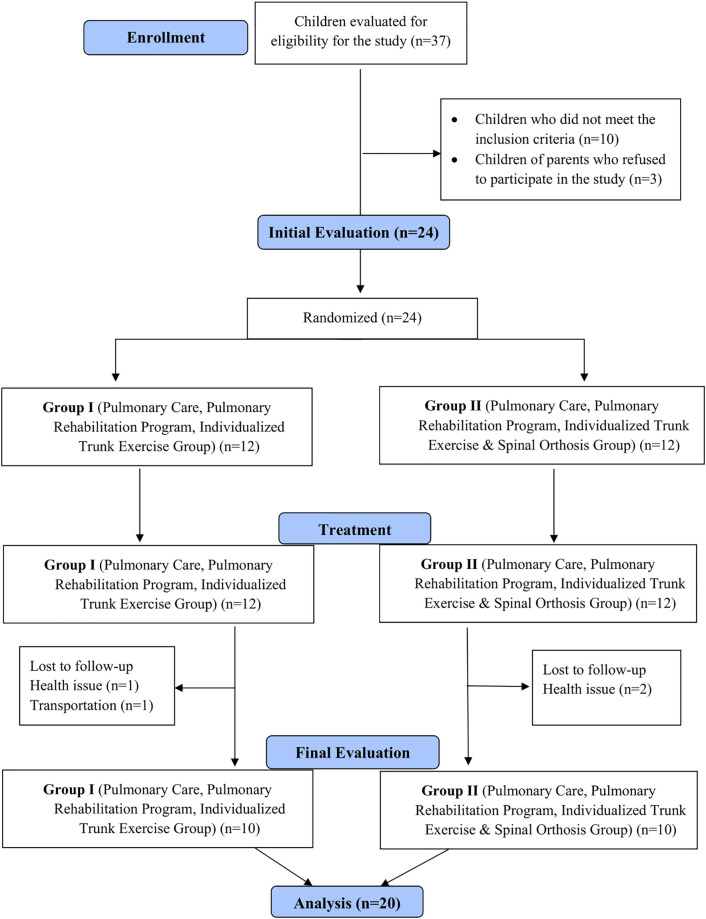
Design and flow chart of the study.

**Fig 2:**
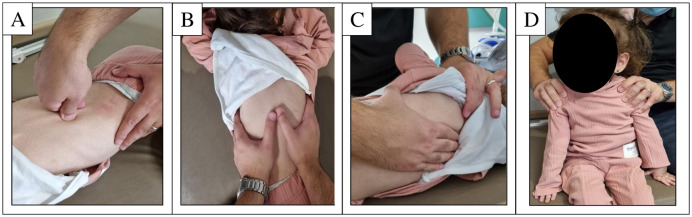
Manual mobilization techniques applied during the pulmonary rehabilitation program: A. Vertebral mobilization, B. Costovertebral mobilization, C. Scapular mobilization, D. Pectoral muscle stretching.

The assessments were conducted based on specific inclusion and exclusion criteria. The study included children between the ages of 2 and 6 who had a confirmed clinical and genetic diagnosis of Type 1 SMA. Participants were required to have scoliosis with a Cobb angle between 20° and 40°, no history of prior spinal surgery, and either ongoing or completed medical treatments with nusinersen and onasemnogene abeparvovec (OA). All patients included in this study had previously received nusinersen treatment before the initiation of gene therapy (OA).

Exclusion criteria included individuals with acute respiratory failure, severe airway infections, or 24-hour mechanical ventilation dependency. Patients receiving medical treatment in an intensive care unit, those with additional orthopedic or neurological conditions, and children at risk of impaired chest expansion or oxygenation who were unable to tolerate orthotic use were also excluded. Furthermore, children and their parents who failed to adhere to the study protocol, including proper use of TLSO, pulmonary care, pulmonary rehabilitation, and trunk exercises, were not included in the study.

A total of 37 patients were evaluated in the study. Following the initial assessments, 13 patients who did not meet the inclusion criteria were excluded. After randomization, 24 patients were enrolled and initiated treatment. However, four patients withdrew from the study before completing the treatment program: two patients from Group 1 (health issue: 1, transportation: 1) and two patients from Group 2 (health issue: 2). At the end of the eight-week treatment program, the study was completed with 20 patients. The CONSORT flow diagram of the study is presented in Fig 1.

#### Randomization and blinding.

After completing the initial assessments for patients who met the inclusion criteria and agreed to participate in the study, a stratified randomization method was applied by a statistician who was not involved in the study to ensure diversity in the case groups. Since Cobb angle (20°-30°, 30°-40°) and age (2–4 years, 4–6 years) are potential factors influencing treatment outcomes, they were used as stratification criteria for randomization. The selection of these stratification criteria was based on prior research demonstrating that these ranges represent clinically significant thresholds for scoliosis progression and therapeutic response in SMA patients. Following the predefined criteria, six strata were formed, each comprising four participants, and sequentially numbered from 1 to 6. Stratum numbers were assigned using the Research Randomizer application (https://www.randomizer.org/). Subsequently, the participants were randomly allocated into two groups, each consisting of 12 children:

Group 1 (Pulmonary Care (PC), Individualized Pulmonary Rehabilitation (IPR), Individualized Trunk Exercise Program (ITE))

Group 2 (Pulmonary Care (PC), Individualized Pulmonary Rehabilitation (IPR), Individualized Trunk Exercise Program (ITE) & TLSO)

### Group 1

Participants received only PC, IPR, and ITE. Parents of the children, who were examined by a specialist physician in the outpatient clinic, were provided with detailed training on PC, IPR, and ITE programs by a experienced physiotherapist, including hands-on practical education. Following the comprehensive briefing provided to the parents regarding all study phases, the intervention phase was initiated. The PC, IPR, and ITE programs were structured as a home-based program, performed once daily, seven days a week, with each session lasting 50–60 minutes over an 8-week period. To track adherence, families were given a weekly follow-up chart. Furthermore, parents and children were invited to the clinic once a week, where the physiotherapist supervised the PC, IPR, and ITE programs, provided guidance to parents, and monitored the child’s progress. This regular follow-up ensured compliance with the home program, allowed real-time adjustments to the rehabilitation program when necessary, and reinforced parental education. The details of the PC, IPR, and ITE programs are provided below.

#### Pulmonary care.

Children diagnosed with Type 1 SMA and referred to the Pediatric Pulmonology Clinic at Istanbul Medipol University Hospital underwent a clinical examination by a specialist physician. Standard clinical decisions were made regarding the necessity, frequency, and settings of Bi-level Positive Airway Pressure (BiPAP) and cough assist device usage as part of routine pulmonary care. A specialist physician established pulmonary care programs for all eligible children before the study began. As a result, all children with Type 1 SMA in the study followed their pre-established pulmonary care protocols, with adjustments made when necessary to BiPAP and cough assist settings and usage frequency.

#### Individualized pulmonary rehabilitation program.

For children diagnosed with Type 1 SMA and referred to our outpatient clinic, routine pulmonary rehabilitation (including inhalation therapy, thoracic vibration, percussion therapy, cough assist device use, secretion aspiration, and postural drainage) was tailored to individual needs following a specialist physician’s clinical assessment. Rehabilitation plans were set before study inclusion, and families were trained accordingly. Additionally, the following manual techniques were implemented as an integral part of the pulmonary rehabilitation program: thoracolumbar and scapular mobilization, as well as pectoral muscle stretching, were incorporated into the rehabilitation program.

Postero-anterior vertebral mobilization was applied in the prone position and tailored to the patient’s motor level. An anterior force was exerted by positioning the thumb and index finger around the spinous process. Similarly, postero-anterior costovertebral mobilization was performed in the prone position, with the thenar region of the hand placed bilaterally on the inferior ribs while applying an anterior force. Each mobilization technique consisted of three repetitions, each lasting one minute. An experienced physiotherapist adjusted the oscillatory force based on the patient’s tolerance level throughout the mobilization process. Scapular mobilization was applied with the patient in a side-lying position. The patient’s arm was supported to relax the surrounding scapular muscles. One hand was placed on the acromion and superior border of the scapula, while the other was positioned on the inferior angle and medial border. Scapular movements were performed in superior, inferior, medial, lateral, upward rotation, downward rotation, and circumduction directions, with each movement repeated 10 times bilaterally. Pectoral muscle stretching was applied in either the supine or long sitting position, depending on the patient’s motor level. Both shoulders were gently retracted, and the stretch was maintained for 10 seconds, repeated 10 times bilaterally. The manual mobilization techniques used during the pulmonary rehabilitation program are illustrated in Fig 2.

#### Individualized trunk exercise program.

**Fig 3:**
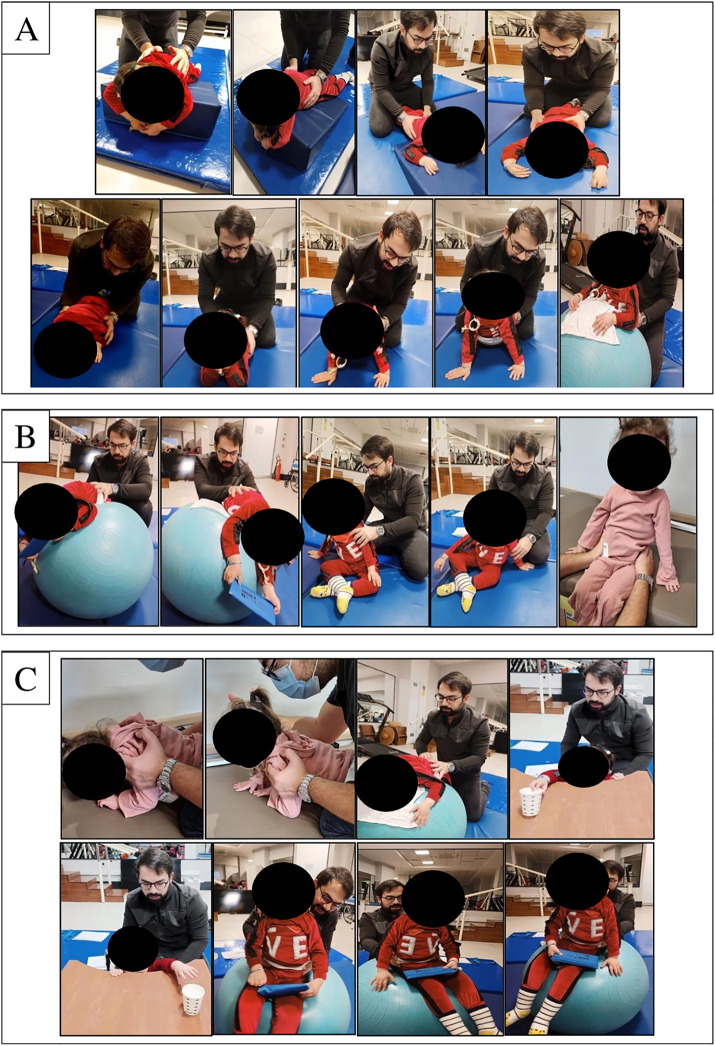
Individualized trunk exercises: A. Trunk extensor exercises, B. Rolling and sitting exercises, C. Weight-shifting exercises.

The exercises were progressed gradually. Each exercise session lasted 20–30 minutes, tailored to the functional levels of the children [27–29] (S1 Table). Examples of the individualized trunk exercises implemented in the study are shown in Fig 3.

### Group 2

Alongside the PC, IPR, and ITE programs implemented for children with Type 1 SMA in Group 2, their families utilized a customized TLSO for at least 8 hours daily over an 8-week period.

#### Thoracolumbosacral orthosis.

**Fig 4:**
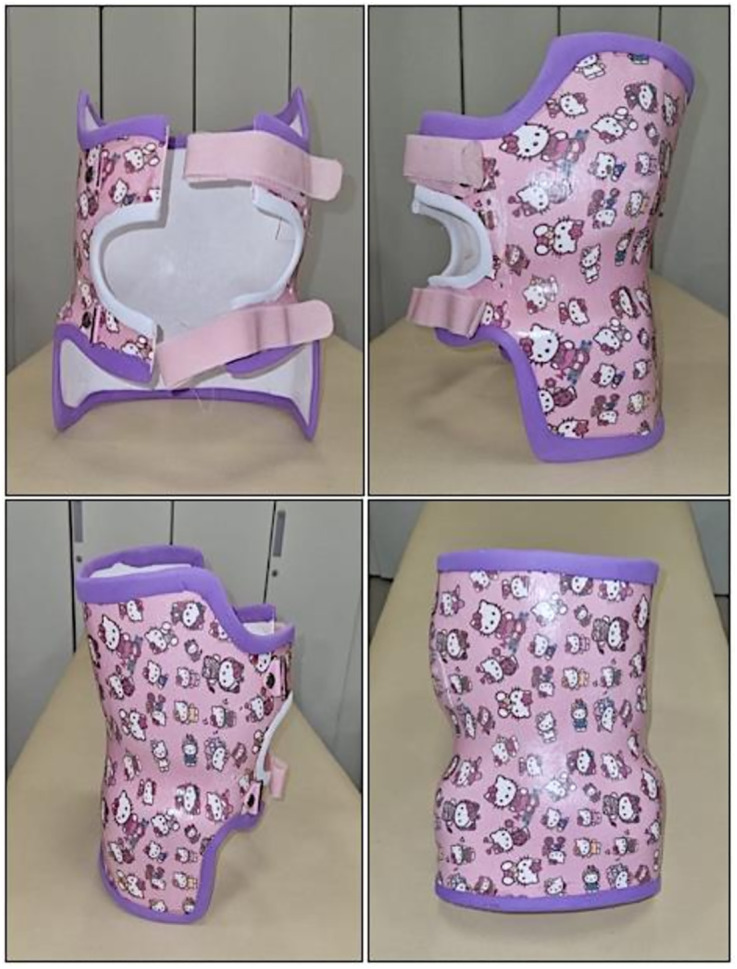
Thoracolumbosacral orthosis.

All orthoses were custom-fabricated by an orthotist-prosthetist. The orthosis design featured an anterior window to facilitate diaphragmatic breathing while ensuring even pressure distribution. This modification allowed for diaphragmatic movement, promoting core muscle strengthening. To support chest expansion, bilateral openings were created between the 4th and 10th thoracic ribs, and a lateral gill modification was incorporated to enhance lower rib mobility. Adjustments were made to the lower anterior section to prevent femoral nerve compression in the seated position, ensuring patient comfort. The inner lining consisted of soft foam, which conformed to the child’s chest anatomy. The orthosis was specifically designed to avoid axillary pressure, with the anterior upper border aligned at the clavicle level and the posterior upper border at the superior scapular margin. This specially designed TLSO provided direct scoliosis correction by stabilizing the iliac crest and ribs, while its advanced vertical suspension system facilitated controlled movement across all planes and promoted an upright spinal posture. The design of the custom-fabricated TLSO is depicted in Fig 4.

Diaphragmatic motion, lateral rib mobility, and apical rib mobility were assessed by an experienced physiotherapist both before orthosis application and after the child had adapted to its use. To ensure physiological safety and monitor potential adverse effects, oxygen saturation (SpO₂) and respiratory rate (RR) were systematically recorded at both time points. During the intervention, oxygen levels were continuously monitored at home by caregivers using a pulse oximeter while the orthosis was being worn.

#### Guidelines for thoracolumbosacral orthosis usage.

− The TLSO should be worn for a minimum of 8 hours per day and should not exceed 10-12 hours. It should be removed every 4-6 hours for ventilation (approximately 20 minutes) and cleaned. This duration should be reduced in hot weather to enhance comfort.
− A seamless cotton undershirt should be worn underneath the orthosis to ensure a proper fit and prevent skin irritation.
− The child’s height and weight should be regularly monitored by the family or caregiver.
− If the orthosis becomes too small due to growth or weight gain, or if signs of redness, bruising, or discomfort appear, the treatment team should be contacted immediately.

Parents recorded the home exercise program and daily TLSO usage duration on a tracking sheet.

### Outcome measures

All measures were assessed at baseline and post-intervention. Two blinded assessors conducted the evaluations. The physician responsible for Cobb angle measurements (a radiologist experienced in spinal imaging) was not involved in the treatment or randomization processes and remained fully blinded to group allocation throughout the study. Each measure was assessed twice, and the final value was the average of both assessments.

#### Cobb angle.

The Cobb angle was determined by measuring the angle on the anteroposterior view of the full-spine X-ray image [10]. To ensure comparability, radiological imaging was performed in the supine position for all patients, regardless of their ability to maintain an unsupported sitting posture [22]. In Group 2, all radiographs were taken out of brace to ensure an accurate evaluation of the natural spinal curvature without orthotic influence. To minimize the transient effects of immediate postural correction, these measurements were conducted 24–48 hours after orthosis removal. Cobb angle measurements were performed using radiographs in the RadiAnt DICOM Viewer (version 3.0.1, Medixant, Poland) by the blinded radiologist, who conducted all Cobb angle measurements independently. The superior and inferior end vertebrae of the spinal curvature were identified. Lines parallel to their endplates were drawn and extended until they intersected. The Cobb angle, representing the degree of spinal curvature, was then measured at this intersection using an angle measurement tool and recorded for further analysis [10].

### Chest deformity

#### Basal upper-lower chest wall ratio.

**Fig 5:**
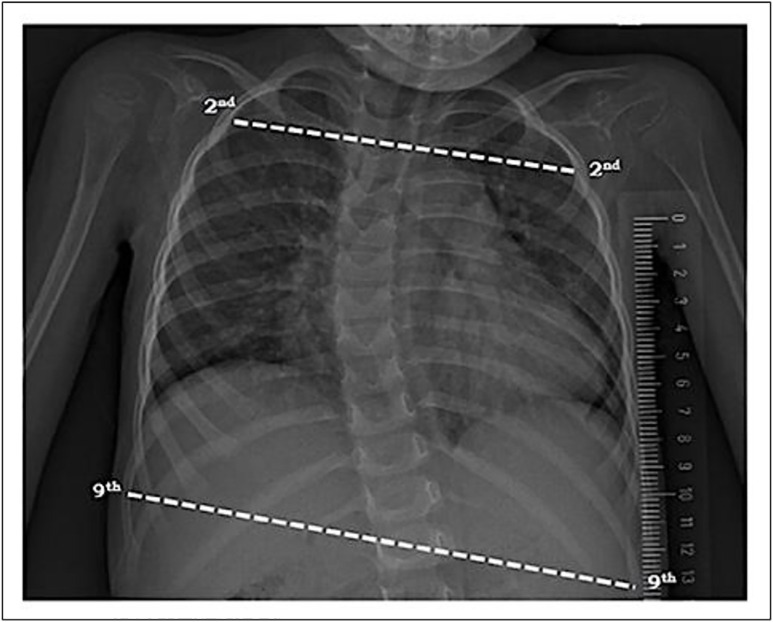
Chest X-ray indicating measurement points for basal upper-lower chest wall ratio.

The basal upper-lower chest wall ratio was evaluated using an anteroposterior chest X-ray taken in the supine position. The same blinded radiologist drew a horizontal reference line from the inner edge of the ribs, and a perpendicular midline was drawn through the spinous processes. The longest line of the 2nd rib (Dapex (upper)) and the 9th rib (Dbase (lower)) was measured. The upper-to-lower chest wall percentage ratio was determined using the formula: Dapex (upper)/ Dbase (lower) × 100 (%). In measuring the bell-shaped chest deformity triggered by impaired thoracic muscles in children with SMA, the bell-shaped chest index, which correlates with the basal upper-to-lower chest ratio, indicates a bell shape when <<1, a rectangular shape when approximately 1, and an inverted triangle shape when>>1 [19,30]. The measurement landmarks used to calculate the basal upper-lower chest wall ratio on chest X-ray are illustrated in Fig 5.

#### Supine angle of trunk rotation.

**Fig 6:**
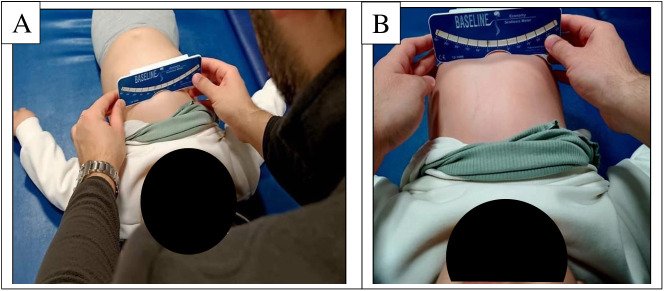
Scoliometer placement on A. upper sternum (SATRU) and B. lower sternum (SATRL) in supine position.

Chest shape was assessed using a Baseline Economy scoliometer. Inside the scoliometer, there is a metal ball that moves within a water bed between a range of 0–30°. The patient was placed in a supine position with the head aligned symmetrically. The scoliometer was placed along the chest at the level of the 2nd-3rd ribs (SATRU (upper)) on the upper part of the sternum and at the lower part of the sternum where the body of the sternum meets the xiphoid process (SATRL (lower)). The measurement was recorded in degrees [17]. The scoliometer placement for SATRU and SATRL measurements is shown in Fig 6.

#### Motor function.

The Children’s Hospital of Philadelphia Infant Test of Neuromuscular Disorders (CHOP INTEND) scale was utilized for the assessment of motor function levels. This 16-item functional evaluation tool has been validated for reliably measuring motor function in infants and young children with Type 1 SMA and other neuromuscular disorders. It is designed for children aged 3 months to over 4 years, though its use is not strictly age-limited. The assessment is structured based on gravity-eliminated, gravity-assisted, and against-gravity movements. Each item is scored on a 0–4 scale, with a maximum possible score of 64. The test is typically completed within 15–40 minutes, incorporating various toys and play objects to facilitate engagement [31]. In addition to CHOP INTEND scoring, patients were also classified into functional motor status categories (non-sitter, sitter, standing with support), based on their highest level of postural control observed during baseline assessments [9].

#### Physiological safety and tolerability during orthosis use.

Physiological safety during TLSO use was evaluated through measurements of RR and SpO_2_. RR was defined as the number of breaths per minute, while SpO_2_ was assessed using a Contec CMS60D handheld pulse oximeter, with the sensor placed on the child’s toe. Measurements were recorded after the orthosis adaptation period to monitor any physiological changes [32]. Tolerability was assessed using the Quebec User Evaluation of Satisfaction with Assistive Technology (QUEST 2.0). Since the participants were children, the questionnaire was completed by their parents during face-to-face interviews. The 12-item scale includes 8 items related to the device and 4 related to the service, each rated on a 5-point Likert scale (1 = not satisfied at all, 5 = very satisfied) [33].

### Statistical analysis

The required sample size was calculated through an a priori power analysis using G*Power software (version 3.0.10). The effect size (Cohen’s d = 1.3) was derived from a study by Catteruccia et al., which reported Cobb angle differences in children with spinal muscular atrophy (SMA) type 2 across three treatment groups (28 ± 10°, 41 ± 16°, and 39 ± 11°, respectively) [22]. This corresponded to a very large effect size (Cohen’s d = 1.3), equivalent to Cohen’s f = 0.65 based on standard conversion formulas. Using this effect size in G*Power (one-tailed independent t-test; α = 0.05; power = 0.80), the required minimum sample size was 18 participants (9 per group). With this sample size, the actual power achieved was 0.8397 (~84%), as transparently reported in the manuscript [34]. To account for potential dropout (~30%), we initially aimed to recruit 24 participants. Ultimately, 20 participants completed the study, preserving adequate statistical power.

Data analysis was performed using SPSS software (version 20; SPSS, Inc., Chicago, IL). Descriptive statistics were expressed as number of units (n), percentage (%), mean, standard deviation (SD), median (M), and minimum (min) and maximum (max) values. Normality of numerical variable distributions was assessed using the Shapiro-Wilk test. Since both descriptive numerical characteristics and variables followed a normal distribution, parametric tests were applied. Categorical variable distributions across groups were analyzed using Chi-square tests (Pearson chi-square/Fisher’s exact test). Between-group differences in numerically measured demographic variables and pre- and post-treatment outcomes were analyzed using the Independent Samples t-test. Within-group pre- and post-treatment comparisons were conducted using the Paired Samples t-test. A p-value of <0.05 was considered statistically significant. Effect sizes were calculated using Cohen’s d, categorized as follows: small (0.20–0.50), medium (0.51–0.80), and large (≥0.81) [35]. The statistician was blinded by group allocation.

## Results

A total of 20 children with Type 1 SMA were included in the study. The demographic and clinical characteristics of the patients, including age, height, weight, gender, number of *SMN2* gene copies, type 1 SMA subtype, age at diagnosis, age at administration of OA, age at first dose of nusinersen, number of nusinersen doses, baseline CHOP INTEND score, motor function status, and Cobb angle were recorded, and it was determined that the two groups had similar demographic and clinical features, including motor function levels (Table 1, p > 0.05). This confirms that the stratified randomization method successfully balanced the baseline characteristics across groups. In the within-group evaluations conducted before and after treatment, significant improvements were observed in the Cobb angle, basal upper-lower chest wall ratio, SATRL, SATRU, and CHOP INTEND parameters for both groups (Table 2, p < 0.05). When the differences between groups before and after treatment were examined, it was found that the Group 2 achieved superior results in all parameters compared to the Group 1 (Cobb angle (ES = 3.98), basal upper-lower chest wall ratio (ES = 5.00), SATRL (ES = 2.55), SATRU (ES = 1.64), and CHOP INTEND (ES = 1.23) (Table 3, p < 0.05). Additionally, to evaluate the physiological safety and tolerability of the orthosis, respiratory rate, oxygen saturation, and QUEST 2.0 satisfaction scores were assessed in Group 2 (Table 4).

**Table 1:** Patients’ demographics and clinical characteristics.

Variables	Group I (mean±SD)	Group II (mean±SD)	p-value
**Age, months** **(Min-Max)**	47.50 ± 11.35 (36.00-64.00)	48.70 ± 11.26 (33.00-63.00)	0.815^a^
**Height, cm** **(Min-Max)**	96.20 ± 9.68 (76.00-106.00)	99.90 ± 7.96 (86.00-112.00)	0.363^a^
**Weight, kg** **(Min-Max)**	12.19 ± 2.42 (7.20-16.00)	12.14 ± 2.34 (8.50-16.50)	0.963^a^
**Gender, F/M (F%)**	4/6 (40%)	6/4 (60%)	0.371^b^
**Number of *SMN2* gene copies,** **2 (%)** **3 (%)**	9 (90%) 1 (10%)	8 (80%) 2 (20%)	1.000^c^
**Age at diagnosis, months** **(Min-Max)**	4.07 ± 3.04 (1.50-12.00)	3.75 ± 1.49 (2.00-6.00)	0.769^a^
**Age at administration of OA, months** **(Min-Max)**	32.40 ± 13.63 (12.00-47.00)	39.60 ± 9.57 (19.00-48.00)	0.189^a^
**Age at 1st dose of nusinersen, months** **(Min-Max)**	7.40 ± 4.59 (1.00-13.00)	6.00 ± 2.30 (3.00-10.00)	0.401^a^
**Number of nusinersen doses** **(Min-Max)**	8.80 ± 2.93 (4.00-14.00)	11.00 ± 2.98 (7.00-15.00)	0.114^a^
**CHOP INTEND**	33.80 ± 19.14 (12.00-62.00)	38.00 ± 18.61 (14.00-62.00)	0,625^a^
**Motor function status,** **Non-sitter (%)** **Sitter (%)** **Standing with support (%)**	6 (60%) 3 (30%) 1 (10%)	6 (60%) 2 (20%) 2 (20%)	0.766^c^
**Cobb Angle, degrees** **(Min-Max)**	28.90 ± 2.76 (24.00-33.00)	29.10 ± 3.78 (23.00-34.00)	0.894^a^

Group 1: PC, IPR, ITE; Group 2: PC, IPR, ITE & TLSO; SD: Standart deviation; Min: Minimum; Max: Maximum; F: Female; M: Male; OA: onasemnogene abeparvovec.

^a^Independent Samples t-test;

^b^Chi-squared test;

^c^Fisher’s exact test.

**Table 2:** Comparison of changes in outcome measures within and between the groups.

Variables	Group I (mean±SD)		p-value	Cohen d (95% CI)	Group II (mean±SD)		p-value	Cohen d (95% CI)
	Pre	Post			Pre	Post		
**Cobb Angle, degrees**	28.90 ± 2.76	27.60 ± 3.09	<0.001	−0.444 (−1.094, 0.206)	29.10 ± 3.78	25.00 ± 3.65	<0.001	−1.103 (−1.889, −0.317)
**Basal upper-lower chest wall ratio**	0.63 ± 0.05	0.66 ± 0.06	<0.001	0.543 (−0.121, 1.207)	0.61 ± 0.06	0.69 ± 0.06	<0.001	1.333 (0.481, 2.185)
**SATRU, degrees**	4.60 ± 2.17	3.80 ± 2.09	<0.001	−0.376 (−1.017, 0.265)	5.90 ± 2.64	3.40 ± 2.31	<0.001	−1.008 (−1.769, −0.247)
**SATRL, degrees**	6.80 ± 1.54	5.40 ± 1.71	<0.001	−0.860 (−1.585, −0.135)	7.50 ± 3.10	4.10 ± 2.18	<0.001	−1.269 (−2.102, −0.436)
**CHOP INTEND**	33.80 ± 19.14	35.50 ± 19.20	<0.001	0.088 (−0.533, 0.709)	38.00 ± 18.61	41.40 ± 18.13	<0.001	0.182 (−0.443, 0.807)

Group 1: PC, IPR, ITE; Group 2: PC, IPR, ITE & TLSO; SATRU: Supine angle of trunk rotation upper; SATRL: Supine angle of trunk rotation lower; CHOP INTEND: The Children’s Hospital of Philadelphia Infant Test of Neuromuscular Disorders; SD: Standart deviation; CI: Confidence interval. Paired Samples t-test.

**Table 3:** Differences within groups before and after treatment and comparison of differences between groups.

Variables	Group I (mean±SD)	Group II (mean±SD)	p-value	Cohen d (95% CI)
**Cobb Angle, degrees**	1.30 ± 0.82	4.10 ± 0.56	<0.001	3.98 (2.46, 5.49)
**Basal upper-lower chest wall ratio**	0.03 ± 0.01	0.08 ± 0.01	<0.001	5.00 (3.22, 6.78)
**SATRU, degrees**	0.80 ± 0.42	2.50 ± 0.84	<0.001	2.55 (1.37, 3.73)
**SATRL, degrees**	1.40 ± 0.51	3.40 ± 1.64	0.002	1.64 (0.62, 2.65)
**CHOP INTEND**	1.70 ± 0.82	3.40 ± 1.77	0.013	1.23 (0.27, 2.18)

Group 1: PC, IPR, ITE; Group 2: PC, IPR, ITE & TLSO; SATRU: Supine angle of trunk rotation upper; SATRL: Supine angle of trunk rotation lower; CHOP INTEND: The Children’s Hospital of Philadelphia Infant Test of Neuromuscular Disorders; SD: Standart deviation; CI: Confidence interval. Independent Samples t-test.

**Table 4:** Physiological safety and tolerability during orthosis use in group 2.

Variables	Respiratory Rate (breaths/minute)	SpO_2_ (%)	QUEST 2.0 Total Score	QUEST 2.0 Device	QUEST 2.0 Service
**mean±SD** **(Min-Max)**	35.40 ± 3.59 (29.00-40.00)	98.50 ± 0.70 (97.00-99.00)	4.53 ± 0.26 (4.06-4.94)	4.61 ± 0.17 (4.37-4.87)	4.45 ± 0.38 (3.75-5.00)

Group 2: PC, IPR, ITE & TLSO; SpO_2_: Oxygen saturation (%); QUEST: Quebec User Evaluation of Satisfaction with Assistive Technology; SD: Standart deviation; Min: Minimum; Max: Maximum.

## Discussion

Upon analyzing the study results, significant improvements were observed in Cobb angle, bell-shaped chest deformity, and motor function in both groups. However, Group 2 exhibited a greater effect size across all assessment parameters. In terms of differences in improvement across Cobb angle, bell-shaped chest deformity, and motor function, Group 2 was found to be superior.

Recent advancements in disease-modifying therapies have significantly improved motor performance, respiratory function, and skeletal development in Type 1 SMA patients [36]. However, these improvements depend not only on pharmacological treatment but also on multidisciplinary management, including orthotic interventions and physiotherapy [37]. Despite ongoing advancements, there is no consensus on the optimal rehabilitation strategies for SMA [36]. Studies highlight the importance of trunk-strengthening and stretching exercises in improving motor function and managing scoliosis in SMA [38]. Additionally, trunk support has been shown to enhance upper extremity performance and postural control, making it a key component of rehabilitation programs [39]. In line with this, both groups in this study followed PC, IPR, and ITE programs, while Group 2 also used a personalized TLSO, which may have contributed to greater postural and functional improvements. A multidisciplinary approach is essential for optimizing therapeutic outcomes in SMA [40]. Effective communication between healthcare providers, patients, and caregivers is crucial to overcoming treatment barriers and ensuring adherence to rehabilitation protocols. Consistent with the literature, all children in this study had their PB programs personalized within a multidisciplinary framework, and families were thoroughly trained by expert physiotherapist for proper implementation.

A review of the literature indicates that scoliosis has historically been a significant issue for patients with Type 2 SMA [13,14,41]. However, in the absence of pharmacological treatment options, the prognosis for infants with Type 1 SMA was generally poor, often leading to early mortality. As a result, spinal deformities in this patient group were rarely documented or remained untreated. Scoliosis remains a major unresolved issue in children with Type 1 SMA. To our knowledge, no studies have assessed the effectiveness of a trunk brace combined with trunk exercises in mitigating scoliosis progression in children diagnosed with Type 1 SMA. To enhance the current understanding of this topic and maintain a homogeneous study population, patients with Type 2 SMA were excluded.

Granata et al. (1974–1988) analyzed spinal radiographs of 63 SMA patients. In intermediate-form SMA (able to sit), scoliosis progressed by 8.3° per year, while in mild-form SMA (lost walking ability), it increased by 2.9° annually. Despite spinal orthosis use for scoliosis >20°, curvature worsened, progressing by 12° per year [13]. Merlini et al. reported scoliosis progression in Type 2 and Type 3 SMA, with annual increases of 8° in Type 2, 3° in non-ambulatory Type 3, and 0.6° in ambulatory Type 3 patients [41]. Rodillo et al. found that scoliosis in intermediate SMA began early, progressed rapidly, and often required spinal fusion. While bracing provided partial correction, it did not halt progression [14]. A study involving 194 SMA patients suggested corsets as a temporary measure for young children before spinal stabilization surgery [42]. Wijngaarde et al. found that scoliosis progressed by 7.2° annually [43]. Catteruccia et al. used Garches corsets to slow scoliosis progression and maintain head-supported sitting. This proactive approach reduced progression to 4.2° per year, improved surgical outcomes, and decreased complications. The study also highlighted the reliability of supine Cobb angle measurements in assessing fixed deformities, eliminating the influence of gravity and muscle strength [22]. Over the past decade, scoliosis incidence in SMA has shifted from Type 2 to Type 1 patients due to new treatments [16,36,44–46]. Al Amrani et al. found rapid scoliosis progression in Type 1 SMA patients treated with nusinersen, with a monthly increase of 2.3° (range: 1.1°–3.8°). Scoliosis developed within the first year in all patients treated before six months, with Cobb angles exceeding 15° by year’s end, showing significantly faster progression than in Type 2 SMA [45]. Kotulska et al. noted that while pharmaceutical treatments improved motor function and survival, early-onset scoliosis remained a concern [44]. Stettner et al. found that 67% of nine pediatric SMA patients treated with OA developed scoliosis, with angles between 20° and 54° after one year, stressing the need for thorough scoliosis evaluation in Type 1 SMA [46]. A retrospective study of 16 SMA patients treated with OA (2020–2022) found significant scoliosis (mean Cobb angle: 24°) in nine cases, indicating early-onset scoliosis as a clinical challenge for SMA children undergoing gene therapy [36]. A study in Turkey involving 29 SMA Type 1 patients (2017–2021) treated with intrathecal nusinersen found scoliosis in 27 patients, all of whom developed scoliosis before age two, with an average Cobb angle of 39.3° and an annual increase of 12.7° [16]. In parallel with these findings, recent real-world data further reinforce the need to revise spinal care protocols in light of evolving SMA phenotypes under disease-modifying treatments. Drain et al. documented a case of a young Type 1 SMA child who received OA gene therapy at five months of age and exhibited rapid scoliosis progression, necessitating magnetic growing rod implantation at just 18 months. This underscores the aggressive course of scoliosis despite early genetic intervention [47]. Similarly, Ip et al. demonstrated that greater motor milestone achievements—such as progression from non-sitter to supported or independent sitter—were paradoxically associated with faster scoliosis progression in Type 1 SMA patients treated with Nusinersen. In contrast, a protective effect was observed in SMA Types 2 and 3, where improved HFMSE scores correlated with slower curvature progression. Their findings indicate that age at treatment initiation, baseline Cobb angle, and motor gains are all critical modifiers of scoliosis risk in this population. Indeed, a young girl with Type 1 SMA from that cohort (patient 2) was reported to be pending spinal surgery at the age of 4 years and 8 months, despite receiving early intervention [48]. Taken together, these emerging data emphasize the urgency of developing targeted, anticipatory spinal management strategies tailored to the unique trajectory of Type 1 SMA under current therapeutic regimens. Our study, consistent with the literature, found significant scoliosis in Type 1 SMA children receiving medical treatment. Radiological imaging in the supine position allowed for comparisons, independent of the ability to maintain a sitting position. After eight weeks, Cobb angles decreased by 1.3° in Group 1 and 4.1° in Group 2, with significant improvement in both. The observed improvement is likely due to the ITE. The effect size was 0.47 for Group 1 (small) and 1.08 for Group 2 (large), suggesting that TLSO use may improve scoliosis prognosis in Type 1 SMA patients.

In SMA patients, special attention should be given to the abdominal area when wearing a corset due to respiratory muscle weakness. Restricting abdominal wall movement affects the diaphragm, and excessive thoracic compression can worsen lung function [49]. In patients with a gastrostomy tube, sufficient space must be provided for both the tube and corset. Regular clinical monitoring is necessary to prevent pressure sores and chest deformities as the body grows, requiring corset adjustments [50]. Di Pede et al. designed a corset with an abdominal opening to allow diaphragm movement in Type 2 SMA patients, incorporating an elastic band to support expiration and enhance abdominal wall response [51]. Similarly, our TLSO design featured windows between the 4th and 10th thoracic ribs to facilitate thoracic expansion. Lateral rib movement was enabled through lateral gill modification, and a front window supported diaphragmatic respiration. This design maintained core strength while allowing diaphragmatic movement. Additionally, the orthosis was adjusted to prevent femoral nerve pressure in the sitting position, lined with soft foam for a comfortable fit, and designed to avoid armpit pressure. It was positioned at clavicle level in the front and at the upper scapula edge in the back, with a TLSO system allowing slight movements in all planes while maintaining vertical spinal alignment through advanced suspension. Bayar et al. examined the short-term effects of an exercise program combined with orthosis use in neuromuscular scoliosis, where a polyethylene spinal orthosis with an anterior opening was worn 8–10 hours daily [52]. Similarly, Kinali et al. recommended that Duchenne muscular dystrophy patients with a Cobb angle >20° wear a polypropylene-molded TLSO for at least 6–8 hours daily while sitting [53]. Catteruccia et al. also suggested a body corset for at least 8 hours, demonstrating effective outcomes [22]. In our study, consistent with the literature, the TLSO was worn for a minimum of 8 hours per day, without exceeding 10–12 hours. Despite concerns in the literature regarding the potential restrictive impact of orthotic bracing on respiratory mechanics (particularly in infants with diaphragmatic weakness) our findings indicated stable respiratory parameters. RR and SpO_2_ remained within normal clinical limits throughout orthosis use [54,55]. In addition, caregiver satisfaction regarding the use of the TLSO was generally found to be high, as assessed by the QUEST 2.0 questionnaire [33]. These findings suggest that, beyond its potential role in scoliosis management, the orthosis was generally well tolerated by the pediatric patient group included in the study. This level of tolerability may be attributed to the individualized design features of the orthosis, which were intended to support diaphragmatic movement and maintain chest wall mobility.

In infants and children with SMA, due to the limited applicability of dynamic pulmonary function tests, bell-shaped chest deformity is commonly assessed using the basal upper-lower chest wall ratio [56,57]. A study found that the bell-shaped chest index correlates with this ratio, classifying chest shapes as bell-shaped (<1), rectangular (~1), and inverted triangle (>1). The study included Type 1, 2, and 3 SMA patients, as well as a healthy control group, all receiving standard care. Bell-shaped chest index values were reported as 0.92 in the healthy group, 0.91 in SMA Type 3, 0.81 in SMA Type 2 without paradoxical breathing, 0.74 in SMA Type 2 with paradoxical breathing (SMA2px), and 0.73 in SMA Type 1. Among 11 SMA2 patients with scoliosis, the upper and lower chest ratios measured from anteroposterior radiographs were 0.68 [57]. In our study, the basal upper-lower chest wall ratios improved significantly in both groups: from 0.63 to 0.66 in group 1 and from 0.61 to 0.69 in group 2. The effect size was 0.60 (medium) for group 1 and 1.33 (large) for group 2, with a statistically significant difference between the groups, where group 2 showed a greater increase. Our findings highlight that individualized physiotherapy programs and TLSO, when combined with pharmacological treatment, are effective in improving bell-shaped chest deformity in children with Type 1 SMA. The greater improvement observed in group 2 underscores the potential benefits of TLSO in this population.

Stępień et al. assessed chest deformities in 21 pharmacologically untreated children with Type 1 SMA (19 with scoliosis) and compared the results with healthy individuals. Using the SATR test in the supine position, they recorded mean SATRL and SATRU values of 5.6° and 6.3°, respectively. The study highlighted more frequent and severe chest deformities in SMA patients, with greater abnormalities in those with scoliosis [17]. Another study reported lower SATRL and SATRU values of 3.3° and 3.6° in 15 untreated Type 1 SMA patients (13 with scoliosis) [38]. Our study demonstrated significant improvement in SATRL and SATRU values in both groups. SATRL decreased from 4.6° to 3.8° in group 1 (effect size: 0.36, small) and from 5.9° to 3.4° in group 2 (effect size: 0.94, large). Similarly, SATRU declined from 6.8° to 5.4° in group 1 (ES: 0.9, large) and from 7.5° to 4.1° in group 2 (ES: 1.09, large). The reduction in group 2 was significantly greater than in group 1, likely due to the addition of TLSO. This study is the first to evaluate TLSO in Type 1 SMA patients with scoliosis, providing valuable insights for future research.

Assessing functional status is essential for evaluating SMA treatments. CHOP INTEND, a validated tool for motor function (MF) assessment in children with muscle weakness, is specifically designed for Type 1 SMA [31,38]. Natural history studies report an initial CHOP INTEND score of ~20 in Type 1 SMA, rarely exceeding 40 [58]. Alves et al. monitored Type 1 SMA patients over 18 months, reporting CHOP INTEND increases of 10.2 points in those receiving only nusinersen (group 1) and 33 points in those transitioning to OA (group 2) [59]. Al Amrani et al. found significant CHOP INTEND improvements in scoliosis patients treated with nusinersen [45]. In our study, CHOP INTEND scores showed significant improvement in both groups, increasing from 33.8 to 35.5 in group 1 and from 38 to 41.4 in group 2. A statistically significant difference was observed between pre- and post-treatment changes across groups. These improvements indicate the beneficial effects of treatments on motor function. However, when compared to long-term follow-up data in the literature, the scores are lower, likely due to differences in patient age, baseline motor function, and follow-up durations. We attribute the observed improvements following eight weeks of treatment to the combined effect of individualized rehabilitation programs and pharmacological treatments.

Key strengths of the study include achieving the targeted sample size (84% power), ensuring a homogeneous patient group by including only individuals with Type 1 SMA, and providing meaningful contributions to the existing literature. Importantly, this is the first study to examine the combined effects of trunk exercises and TLSO interventions in children with Type 1 SMA and scoliosis. The treatment of all participants was delivered by a single experienced physiotherapist, which ensured consistency and standardization of the exercise protocol. However, several limitations should be acknowledged. First, although the initial plan was to evaluate existing spinal X-rays, early Cobb angle measurements (before six months) in some patients were not available and should be considered a limitation. Second, the lack of full blinding may have introduced potential performance or detection bias, reducing the internal validity of the results. Third, detailed data on patients’ daily postural positioning were not systematically recorded. Given that gravitational spinal loading may vary significantly based on daily positioning, particularly in non-sitter children who are frequently placed in supportive seating, this represents a potentially relevant confounding factor that may have influenced scoliosis progression. Additionally, due to ethical considerations regarding cumulative radiation exposure in pediatric patients, especially those with SMA, follow-up spinal radiographs were not obtained within the first 1–3 months post-intervention. To minimize unnecessary radiation burden, Cobb angle measurements were limited to pre- and post-intervention time points.

The improvements observed in this study should be interpreted as short-term outcomes. Future research should investigate the long-term impact of the Group 2 intervention to confirm its sustained clinical benefits. Nevertheless, given the promising outcomes observed over the 8-week intervention, this protocol appears to be suitable for children with SMA aged 2–6 years presenting with a Cobb angle greater than 20°, using TLSO in accordance with recommended daily wear duration. A prospective follow-up protocol has been initiated to assess the durability of the observed outcomes using longer-term imaging and functional evaluations.

## Conclusions

This study demonstrated that the use of TLSO combined with ITE is effective in reducing scoliosis progression, improving chest wall configuration, and enhancing motor function in children with Type 1 SMA. Current clinical guidelines generally recommend TLSO use in patients with a Cobb angle greater than 20°, and our findings support this threshold. Furthermore, our results suggest that initiating orthotic treatment proactively in the early stages of scoliosis may help prevent further curvature progression. The addition of TLSO to standard care involving PC, IPR, and ITE yielded superior outcomes in terms of scoliosis progression, chest deformity, and motor function. These findings highlight the clinical value of a multidisciplinary early intervention approach that includes spinal mobilization, trunk strengthening, and pulmonary exercise components for the effective management of scoliosis in children with Type 1 SMA. Therefore, clinicians should consider incorporating TLSO into the standard rehabilitation program for children with Type 1 SMA and Cobb angles >20°, as part of a comprehensive early intervention strategy.

## Supporting information

S1 Table Individualized trunk exercise program. (DOCX)

S2 Table Baseline clinical characteristics of all recruited patients at study entry. (DOCX)

S1 Data CONSORT Checklist. (DOC)

S2 Data Study protocol. (PDF)
