# Supplementary material for: The effect of thoracolumbosacral orthosis on scoliosis progression and chest deformity in children with type 1 spinal muscular atrophy: A randomized controlled trial
Source: PLoS One. 2025 Sep 15;20(9):e0323341. doi: 10.1371/journal.pone.0323341 (PMC12435727; doi:10.1371/journal.pone.0323341)
Supplement: S1 Table — (DOCX) [file pone.0323341.s001.docx]

**Individualized Trunk Exercise Program**

|  | **Exercise Level** | | |  |
| --- | --- | --- | --- | --- |
| **Exercises** | **Beginner** | **Intermediate** | **Advanced** | **Frequency** |
| **Trunk Extensor Exercises** | Lifting the head and trunk from a prone position on an inclined firm surface | Lifting the head and trunk from a prone position on a reduced-incline firm surface | Lifting the head and trunk from a prone position on a flat firm surface | 1 set, 10 repetitions |
|  | Lifting the head and trunk from a prone position on an inclined soft surface | Lifting the head and trunk from a prone position on a reduced-incline soft surface | Lifting the head and trunk from a prone position on a flat soft surface | 1 set, 10 repetitions |
|  | Lifting the head and trunk from a prone position on an inclined firm surface and holding for 5 seconds | Lifting the head and trunk from a prone position on a reduced-incline firm surface and holding for 5 seconds | Lifting the head and trunk from a prone position on a flat firm surface and holding for 5 seconds | 1 set, 10 repetitions |
|  | Lifting the head and trunk from a prone position on an inclined soft surface and holding for 5 seconds | Lifting the head and trunk from a prone position on a reduced-incline soft surface and holding for 5 seconds | Lifting the head and trunk from a prone position on a flat soft surface and holding for 5 seconds | 1 set, 10 repetitions |
|  | Transitioning the shoulders and elbows to a 90-90 position from a prone position on a firm surface | Transitioning the shoulders and elbows to a 90-90 position from a prone position on a soft surface | Performing trunk extension while the patient’s trunk is positioned on a swiss ball | 1 set, 10 repetitions |
| **Rolling and Sitting Exercises** | Rolling activity in a prone position on a firm surface | Rolling activity in a prone position on a soft surface | Rolling activity in a prone position on a swiss ball | 1 set, 10 repetitions (right-left) |
|  | Rolling activity in a supine position on a firm surface | Rolling activity in a supine position on a soft surface | Rolling activity in a supine position on a swiss ball | 1 set, 10 repetitions (right-left) |
|  | Transitioning from a supine position to a sitting position on an inclined firm surface | Transitioning from a supine position to a sitting position on a reduced-incline firm surface | Transitioning from a supine position to a sitting position on a flat firm surface | 1 set, 10 repetitions (right-left) |
| **Weight-Shifting Exercises** | Weight-shifting activity in a prone position with shoulders and elbows at 90-90 on a firm surface (front-back and right-left) | Weight-shifting activity in a prone position with shoulders and elbows at 90-90 on a soft surface (front-back and right-left) | Weight-shifting activity in a prone position with shoulders and elbows at 90-90 on a swiss ball (front-back and right-left) | 1 set, 10 repetitions (right-left) |
|  | Reaching activity in a prone position on a firm surface toward a target placed at eye level and within the patient's reachable distance | Reaching activity in a prone position on a soft surface toward a target placed at eye level and within the patient's reachable distance | Reaching activity in a prone position on a swiss ball toward a target placed at eye level and within the patient's reachable distance | 1 set, 10 repetitions (right-left) |
|  | Reaching activity while sitting on a firm surface toward an object placed on a table in front of the patient | Reaching activity while sitting on a soft surface toward an object placed on a table in front of the patient | Reaching activity while sitting on a swiss ball or balance board toward an object placed on a table in front of the patient | 1 set, 10 repetitions |
|  | Reaching activity while sitting on a firm surface toward objects placed on tables positioned to the right and left of the patient | Reaching activity while sitting on a soft surface toward objects placed on tables positioned to the right and left of the patient | Reaching activity while sitting on a Swiss ball or balance board toward objects placed on tables positioned to the right and left of the patient | 1 set, 10 repetitions |
