# Supplementary material for: The effect of thoracolumbosacral orthosis on scoliosis progression and chest deformity in children with type 1 spinal muscular atrophy: A randomized controlled trial
Source: PLoS One. 2025 Sep 15;20(9):e0323341. doi: 10.1371/journal.pone.0323341 (PMC12435727; doi:10.1371/journal.pone.0323341)
Supplement: S2 Table — (DOCX) [file pone.0323341.s002.docx]

**Baseline clinical characteristics of all recruited patients at study entry**

| **Patient ID** | **Group** | **Age (months)** | **SMA Treatment Type** | ***SMN2* Copy Number** | **Age at Initiation of Nusinersen (months)** | **Age at Initiation of Gene Therapy (months)** | **Motor Status** | **CHOP INTEND Score** | **Cobb Angle (degrees)** | **Ventilation Support** | **Feeding Support** | **Nusinersen Before Gene Therapy** |
| --- | --- | --- | --- | --- | --- | --- | --- | --- | --- | --- | --- | --- |
| P1 | 1 | 36 | Nusinersen + Gene therapy | 2 | 4 | 22 | Non-sitter | 38 | 24 | BiPAP at night | PEG tube | Yes |
| P2 | 1 | 43 | Nusinersen + Gene therapy | 2 | 6 | 32 | Non-sitter | 12 | 26 | No support | PEG tube | Yes |
| P3 | 1 | 39 | Nusinersen + Gene therapy | 2 | 3 | 12 | Non-sitter | 14 | 29 | No support | PEG tube | Yes |
| P4 | 1 | 37 | Nusinersen + Gene therapy | 2 | 3 | 14 | Non-sitter | 18 | 29 | No support | PEG tube | Yes |
| P5 | 1 | 52 | Nusinersen + Gene therapy | 2 | 12 | 45 | Non-sitter | 23 | 31 | No support | PEG tube | Yes |
| P6 | 1 | 40 | Nusinersen + Gene therapy | 2 | 8 | 23 | Sitter | 36 | 30 | No support | Oral feeding | Yes |
| P7 | 1 | 64 | Nusinersen + Gene therapy | 2 | 13 | 46 | Sitter | 55 | 33 | No support | Oral feeding | Yes |
| P8 | 1 | 62 | Nusinersen + Gene therapy | 3 | 13 | 47 | Standing with support | 59 | 31 | BiPAP at night | Oral feeding | Yes |
| P9 | 1 | 62 | Nusinersen + Gene therapy | 2 | 11 | 42 | Non-sitter | 21 | 26 | No support | PEG tube | Yes |
| P10 | 1 | 40 | Nusinersen + Gene therapy | 2 | 1 | 41 | Sitter | 62 | 30 | BiPAP at night | NG tube | Yes |
| P11 | 2 | 61 | Nusinersen + Gene therapy | 2 | 8 | 41 | Non-sitter | 18 | 24 | No support | PEG tube | Yes |
| P12 | 2 | 62 | Nusinersen + Gene therapy | 2 | 6 | 48 | Sitter | 55 | 33 | BiPAP at night | NG tube | Yes |
| P13 | 2 | 63 | Nusinersen + Gene therapy | 2 | 8 | 37 | Non-sitter | 23 | 30 | BiPAP at night | NG tube | Yes |
| P14 | 2 | 46 | Nusinersen + Gene therapy | 3 | 10 | 19 | Standing with support | 62 | 27 | No support | Oral feeding | Yes |
| P15 | 2 | 55 | Nusinersen + Gene therapy | 2 | 3 | 38 | Non-sitter | 35 | 32 | BiPAP at night | PEG tube | Yes |
| P16 | 2 | 33 | Nusinersen + Gene therapy | 2 | 6 | 48 | Non-sitter | 14 | 23 | No support | NG tube | Yes |
| P17 | 2 | 47 | Nusinersen + Gene therapy | 2 | 4 | 45 | Non-sitter | 36 | 27 | BiPAP at night | PEG tube | Yes |
| P18 | 2 | 47 | Nusinersen + Gene therapy | 2 | 3 | 45 | Sitter | 54 | 32 | BiPAP at night | Oral feeding | Yes |
| P19 | 2 | 39 | Nusinersen + Gene therapy | 2 | 5 | 28 | Non-sitter | 22 | 34 | No support | Oral feeding | Yes |
| P20 | 2 | 34 | Nusinersen + Gene therapy | 3 | 7 | 47 | Standing with support | 61 | 29 | BiPAP at night | Oral feeding | Yes |

Group 1: PC, IPR, ITE; Group 2: PC, IPR, ITE & TLSO; SMA: Spinal Muscular Atrophy; CHOP INTEND: Children’s Hospital of Philadelphia Infant Test of Neuromuscular Disorders; BiPAP: Bilevel Positive Airway Pressure; PEG tube: Percutaneous Endoscopic Gastrostomy tube; NG tube: Nasogastric tube.
