# Supplementary material for: The effect of thoracolumbosacral orthosis on scoliosis progression and chest deformity in children with type 1 spinal muscular atrophy: A randomized controlled trial
Source: PLoS One. 2025 Sep 15;20(9):e0323341. doi: 10.1371/journal.pone.0323341 (PMC12435727; doi:10.1371/journal.pone.0323341)
Supplement: S2 Data — (PDF) [file pone.0323341.s004.pdf]

STUDY PROTOCOL

The Effect of Spinal Orthosis on Scoliosis Progression and Chest Deformity in Children with Type 1 Spinal Muscular Atrophy: A Randomized Controlled Trial

1. Introduction

Spinal muscular atrophy (SMA) is an autosomal recessive neuromuscular disease caused by the deletion of the SMN1 gene located on chromosome 5, which is responsible for the production of the Survival Motor Neuron (SMN) protein. Altered production of this protein leads to degeneration of motor neurons in the anterior horn of the spinal cord, resulting in progressive denervation, muscle atrophy, and increased weakness.

After cystic fibrosis, SMA is the second most common autosomal recessive genetic disease of childhood, with a global incidence of 1/10,000 to 1/24,000 live births. The carrier frequency in the general population is approximately 1/40 to 1/60 (Di Pede et al., 2019).

SMA includes a wide range of phenotypes classified into clinical groups according to age of onset and maximum motor function achieved:

- Very weak infants who cannot sit independently (Type 1),
- Patients who can sit independently but cannot stand (Type 2),
- Ambulatory patients with childhood-onset SMA (Type 3),
- Ambulatory patients with adult-onset SMA (Type 4) (Mercuri et al., 2018).

Type 1 SMA (Werdnig-Hoffmann disease, infantile-onset) appears before 6 months of age and is the most common type of SMA (60%). These patients are severely hypotonic, with predominant proximal muscle weakness, weak cry, respiratory failure or abdominal breathing, difficulties in sucking or swallowing, tongue fasciculations, and high risk of aspiration pneumonia. They never learn to sit and, in the absence of ventilatory support, typically die from respiratory failure within the first two years of life (Mirea et al., 2022).

In these patients, intercostal muscle weakness and relative preservation of the diaphragm result in a characteristic "bell-shaped" chest and paradoxical breathing pattern (Mercuri et al., 2018). Scoliosis, defined by the Scoliosis Research Society as a three-dimensional spinal deformity with a Cobb angle of at least 10°, is most commonly observed in patients with Type 1 and Type 2 SMA (Stępień et al., 2020). Scoliosis significantly impairs pulmonary function by reducing vital capacity and increasing ventilation/perfusion mismatch (Di Pede et al., 2019). Thoracic cage distortion related to scoliosis further exacerbates respiratory disorders in SMA patients (Stępień et al., 2020). Cobb angle measured in supine position or with spinal orthosis in sitting position can be used for follow-up (Sauvagnac-Quera et al., 2016).

The advent of new treatments leading to increased survival and overall functional improvement is rapidly changing the scenario of spinal management in these patients (Finkel et al., 2018). For SMA Types 1 and 2, scoliosis >20° should be monitored every 6 months until skeletal maturity and annually thereafter. Spinal orthoses are frequently recommended to support the hypotonic trunk and manage scoliosis >20° (Fujak et al., 2011; Catteruccia et al., 2015).

Since both rigid and soft thoracolumbosacral orthoses have been recommended, there is no consensus regarding the optimal brace type (Mercuri et al., 2018). No studies have been found in the literature specifically evaluating the effects of spinal orthosis in patients with Type 1 SMA. The aim of this study is to investigate the effect of spinal orthosis on scoliosis progression and chest deformity in children with Type 1 SMA.

2. Objectives

- **Primary Objective:** To assess the effect of spinal orthosis on scoliosis progression, as measured by the Cobb angle.
- **Secondary Objectives:**
  - To evaluate changes in bell-shaped chest deformity using the basal upper-lower chest wall ratio.
  - To assess improvements in motor function using the CHOP INTEND scale.
  - To compare the effects of spinal orthosis combined with individualized trunk exercises versus trunk exercises alone.

3. Methods

3.1. Study Design

This study is a stratified, single-blind, parallel-group randomized controlled trial conducted at Istanbul Medipol University Hospital. Recruitment will begin on October 11, 2023, and is expected to conclude by September 10, 2024.

- **Group 1** (Pulmonary Care (PC), Individualized Pulmonary Rehabilitation (IPR), Individualized Trunk Exercise Program (ITE))
- **Group 2** (Pulmonary Care (PC), Individualized Pulmonary Rehabilitation (IPR), Individualized Trunk Exercise Program (ITE) & Spinal Orthosis)

3.2 .Ethical Considerations

Ethical approval was obtained from the Non-Interventional Ethics Committee of Istanbul Medipol University (Approval No: E-10840098-772.02-200). The study is registered in ClinicalTrials.gov (NCT05878418). Written informed consent will be obtained from all participants before enrollment. All procedures adhere to the Declaration of Helsinki.

3.3. Participant Selection

Inclusion Criteria:

- Children aged 2–6 years with a genetically confirmed diagnosis of Type 1 SMA.
- Cobb angle between 20° and 40°.
- Undergoing or completed treatment with nusinersen and onasemnogene abeparvovec (OA).

Exclusion Criteria:

- Acute respiratory failure or mechanical ventilation dependency.
- Previous spinal surgery.
- Additional neurological or orthopedic disorders.
- Poor adherence to spinal orthosis or exercise protocols.

3.4. Randomization & Blinding

Stratified randomization based on Cobb angle and age will be performed using Research Randomizer. Six strata will be created. A statistician blinded to group allocation will conduct the randomization. Assessors will be blinded.

3.5. Intervention Details

3.5.1. Group 1:

Participants will receive an individualized trunk exercise (ITE) program along with standard pulmonary care (PC) and pulmonary rehabilitation (IPR). Exercises will be performed daily for 8 weeks, under weekly clinical supervision.

Exercises include:

- Passive and active-assisted trunk mobilization exercises.
- Core stability training through guided postural activities.
- Supine and side-lying activities to enhance trunk control.
- Gentle active resistance exercises for trunk muscles.
- Breathing exercises to enhance diaphragmatic movement.

3.5.2. Group 2:

- Participants will receive the same intervention as Group 1, with the addition of a customized thoracolumbosacral orthosis (TLSO) worn for at least 8 hours daily.

  The TLSO will feature:

- An anterior window for diaphragmatic breathing.
- Lateral modifications to facilitate chest expansion.
- Soft inner padding for enhanced comfort.
- Adjustable straps to optimize spinal alignment without over-restricting movement.

3.6. Outcome Measures

Assessments will be conducted at baseline (pre-treatment) and after eight weeks of rehabilitation.

Primary Outcome Measure:

• Cobb angle will be assessed using supine spinal X-ray.

Secondary Outcome Measures:

• The basal upper-lower chest wall ratio will be evaluated using chest X-ray.

• Supine Angle of Trunk Rotation (SATRL and SATRU) will be measured using a scoliometer.

• Motor function will be assessed using the CHOP INTEND scale.

• Respiratory rate and SpO_2 will be measured using pulse oximetry.

• Satisfaction with orthosis use will be evaluated using the QUEST 2.0 questionnaire.

3.7. Sample Size Calculation

A priori power analysis conducted using G*Power 3.1 ($f = 0.65$, $\alpha = 0.05$, power = 0.80) will indicate that a minimum of 18 participants (9 per group) will be required. Considering a potential dropout rate of 30%, 24 children will be recruited.

3.8. Statistical Analysis

SPSS version 25.0 will be used. Normality will be assessed using the Shapiro-Wilk test.

- Descriptive statistics: Mean ± SD, median, and range.
- Group comparisons: Independent Samples t-test.
- Within-group comparisons: Paired Samples t-test.
- Effect size calculation: Cohen's d (small: 0.20-0.50, medium: 0.51-0.80, large: ≥0.81).
- Statistical significance: $p < 0.05$.

4. Expected Outcomes

- The combination of spinal orthosis and trunk exercises is expected to lead to greater improvements in scoliosis progression, chest deformity, and motor function compared to trunk exercises alone.
- The Cobb angle is anticipated to show a slower rate of progression in the spinal orthosis group.
- Patients in the spinal orthosis group may demonstrate improved respiratory function due to enhanced chest wall stability.

- The CHOP INTEND scores are expected to improve more significantly in the spinal orthosis group due to better postural control and spinal alignment.

5. Dissemination Plan

Findings will be disseminated through peer-reviewed journal publications, conference presentations, and clinical practice guidelines for physiotherapists managing Type 1 SMA.

6. Conclusion

This study is the first to assess the combined effects of spinal orthosis and individualized trunk exercises on scoliosis progression and chest deformity in children with Type 1 SMA. The results will contribute to evidence-based rehabilitation strategies for managing scoliosis in SMA.

7. References

Catteruccia, M., Vuillerot, C., Vaugier, I., Leclair, D., Azzi, V., Viollet, L., … Quijano-Roy, S. (2015). Orthopedic Management of Scoliosis by Garches Brace and Spinal Fusion in SMA Type 2 Children. *Journal of Neuromuscular Diseases*, *2*(4), 453–462. https://doi.org/10.3233/JND-150084

Davis, S. E., Hynan, L. S., Limbers, C. A., Andersen, C. M., Greene, M. C., Varni, J. W., & Iannaccone, S. T. (2010). The PedsQL^{TM} in pediatric patients with duchenne muscular dystrophy: Feasibility, reliability, and validity of the pediatric quality of life inventory neuromuscular module and generic core scales. *Journal of Clinical Neuromuscular Disease*, *11*(3), 97–109. https://doi.org/10.1097/CND.0b013e3181c5053b

Di Pede, C., Salamon, E., Mottaotta, M., Agostoosto, C., Benini, F., & Ferrari, A. (2019). Spinal bracing and lung function in type-2 spinal muscular atrophy. *European Journal of Physical and Rehabilitation Medicine*, *55*(4), 505–509. https://doi.org/10.23736/S1973-9087.18.05046-3

Finkel, R. S., Mercuri, E., Meyer, O. H., Simonds, A. K., Schroth, M. K., Graham, R. J., … Sejersen, T. (2018). Diagnosis and management of spinal muscular atrophy: Part 2: Pulmonary and acute care; medications, supplements and immunizations; other organ systems; and ethics. *Neuromuscular Disorders*, *28*(3), 197–207. https://doi.org/10.1016/j.nmd.2017.11.004

Fujak, A., Kopschina, C., Forst, R., Mueller, L. A., & Forst, J. (2011). Use of orthoses and orthopaedic technical devices in proximal spinal muscular atrophy. Results of survey in 194 SMA patients. *Disability and Rehabilitation: Assistive Technology*, *6*(4), 305–311. https://doi.org/10.3109/17483107.2010.525292

Glanzman, A. M., Mazzone, E., Main, M., Pelliccioni, M., Wood, J., Swoboda, K. J., … Finkel, R. S. (2010). The Children's Hospital of Philadelphia Infant Test of Neuromuscular Disorders (CHOP INTEND): Test development and reliability The CHOP INTEND is a reliable measure of motor skills in patients with SMA-I and neuromuscular disorders presenting in infancy. *Neuromuscul Disord*, *20*(3), 155–161. https://doi.org/10.1016/j.nmd.2009.11.014.

LoMauro A, Banfi P, Mastella C, Alberti K, Baranello G, Aliverti A. A New Method for Measuring Bell-Shaped Chest Induced by Impaired Ribcage Muscles in Spinal Muscular Atrophy Children. Front Neurol. 2018;9: 703. doi:10.3389/fneur.2018.00703

Mercuri, E., Finkel, R. S., Muntoni, F., Wirth, B., Montes, J., Main, M., … Szlagatys-Sidorkiewicz, A. (2018). Diagnosis and management of spinal muscular atrophy: Part 1: Recommendations for diagnosis, rehabilitation, orthopedic and nutritional care. *Neuromuscular Disorders*, *28*(2), 103–115. https://doi.org/10.1016/j.nmd.2017.11.005

Mirea, A., Leanca, M. C., Onose, G., Sporea, C., Padure, L., Shelby, E. S., … Daia, C. (2022). Physical Therapy and Nusinersen Impact on Spinal Muscular Atrophy Rehabilitative Outcome. *Frontiers in Bioscience - Landmark*, *27*(6). https://doi.org/10.31083/j.fbl2706179

O'Hagen, J. M., Glanzman, A. M., McDermott, M. P., Ryan, P. A., Flickinger, J., Quigley, J., … De Vivo, D. C. (2007). An expanded version of the Hammersmith Functional Motor Scale for SMA II and III patients. *Neuromuscular Disorders*, *17*(9–10), 693–697. https://doi.org/10.1016/j.nmd.2007.05.009

Radiolo-, P., & Johns, T. (1987). I1, *10*(10), 904–906.

Sauvagnac-Quera, R., Vabre, C., Azzi, V., Tirolien, S., Leiba, N., Poisson, F., … Quijano-Roy, S. (2016). Prevention and treatment of scoliosis by Garches Brace in children with type Ib SMA. *Annals of Physical and Rehabilitation Medicine*, *59*, e92.https://doi.org/10.1016/j.rehab.2016.07.207

Stępień, A., Mazurkiewicz, Ł., Maślanko, K., Rekowski, W., & Jędrzejowska, M. (2020). Cervical rotation, chest deformity and pelvic obliquity in patients with spinal muscular atrophy. *BMC Musculoskeletal Disorders*, *21*(1), 1–11. https://doi.org/10.1186/s12891-020-03710-7

Yakut, Y., Yurt, Y., Yağci, G., & Şimşek, İ. E. (2020). EXERCISE THERAPY AND REHABILITATION Quebec Yardımcı Teknoloji Kullanıcı Memnuniyeti Değerlendirme 2.0 Anketi'nin protez ve ortez kullanan bireylerde Türkçe adaptasyonu. *Journal of Exercise Therapy and Rehabilitation*, *7*(3), 284–295. Retrieved from www.jetr.org.tr

Funding

This study was funded by the Scientific and Technological Research Council of Turkey (TUBITAK) under project code 323S007. The funders had no role in the study design, data collection, analysis, or publication decisions.

Competing Interests

The authors declare no competing interests.
